# Tropical Diseases in Travelers

**DOI:** 10.3201/eid1511.091287

**Published:** 2009-11

**Authors:** Lin H. Chen

**Affiliations:** Mount Auburn Hospital, Cambridge, Massachusetts, USA

**Keywords:** Tropical disease, travel medicine, travelers, travelers’ health, book review

This book captures the essence of tropical medicine for clinicians evaluating returning travelers. The editor, an international expert in tropical and travel medicine, authored or coauthored many chapters of the book. The book also reflects the experience of numerous experts in the field of travel medicine.

The book consists of 43 chapters organized into 3 sections: general aspects of tropical diseases in travelers, specific infections, and approaches to specific syndromes. The first section describes general trends in travel medicine and discusses types of studies encountered in travel medicine research. This section provides a basis for screening travelers and makes recommendations for doing so.

The section on specific diagnoses dedicates a chapter each to the most commonly encountered groups of microbial organisms. This section emphasizes the epidemiology of travel illnesses and clinical signs and symptoms in travelers, especially aspects of illness different from those of populations residing in the disease-endemic areas. This section also includes photographs of physical findings in travelers; the photographs highlight such diseases as African tick-bite fever, chikungunya, dengue, swimmer’s itch, African trypanosomiasis, leishmaniasis, measles, tungiasis, and cutaneous larva migrans.

The section on syndromes focuses on approaches to evaluating major complaints in returning travelers. Complaints discussed include posttravel diarrhea, fever, skin problems, eosinophilia, respiratory complaints, rheumatologic conditions, and neurologic findings.

For clinicians, adequate knowledge of illnesses associated with travel is critical to the ability to provide proper pretravel advice. This book contributes much information to assist in understanding diseases encountered by travelers. It is a valuable reference on tropical and travel medicine and is especially important to clinicians managing ill travelers. However, it also supplies fundamental background information for clinicians providing only pretravel consultations. The authors present concise, solid evidence and practical insights on tropical diseases in travelers. I recommend it highly to clinicians involved in the care of travelers in industrialized and developing countries.

